# New Perspectives on Sex Steroid and Mineralocorticoid Receptor Signaling in Cardiac Ischemic Injury

**DOI:** 10.3389/fphys.2022.896425

**Published:** 2022-06-29

**Authors:** Laura A. Bienvenu, James R. Bell, Kate L. Weeks, Lea M. D. Delbridge, Morag J. Young

**Affiliations:** ^1^ Molecular Imaging and Theranostics Laboratory, Baker Heart and Diabetes Institute, Melbourne, VIC, Australia; ^2^ Baker Department of Cardiometabolic Health, University of Melbourne VIC, Melbourne, VIC, Australia; ^3^ Department of Microbiology, Anatomy, Physiology and Pharmacology, La Trobe University, Bundoora, VIC, Australia; ^4^ Department of Anatomy and Physiology, University of Melbourne, Parkville, VIC, Australia; ^5^ Cardiovascular Endocrinology Laboratory, Baker Heart and Diabetes Institute, Melbourne, VIC, Australia

**Keywords:** cardiomyocyte, ischemia-reperfusion, mineralocorticoid, sex differences, nitric oxide

## Abstract

The global burden of ischemic heart disease is burgeoning for both men and women. Although advances have been made, the need for new sex-specific therapies targeting key differences in cardiovascular disease outcomes in men and women remains. Mineralocorticoid receptor directed treatments have been successfully used for blood pressure control and heart failure management and represent a potentially valuable therapeutic option for ischemic cardiac events. Clinical and experimental data indicate that mineralocorticoid excess or inappropriate mineralocorticoid receptor (MR) activation exacerbates ischemic damage, and many of the intracellular response pathways activated in ischemia and subsequent reperfusion are regulated by MR. In experimental contexts, where MR are abrogated genetically or mineralocorticoid signaling is suppressed pharmacologically, ischemic injury is alleviated, and reperfusion recovery is enhanced. In the chronic setting, mineralocorticoid signaling induces fibrosis, oxidative stress, and inflammation, which can predispose to ischemic events and exacerbate post-myocardial infarct pathologies. Whilst a range of cardiac cell types are involved in mineralocorticoid-mediated regulation of cardiac function, cardiomyocyte-specific MR signaling pathways are key. Selective inhibition of cardiomyocyte MR signaling improves electromechanical resilience during ischemia and enhances contractile recovery in reperfusion. Emerging evidence suggests that the MR also contribute to sex-specific aspects of ischemic vulnerability. Indeed, MR interactions with sex steroid receptors may differentially regulate myocardial nitric oxide bioavailability in males and females, potentially determining sex-specific post-ischemic outcomes. There is hence considerable impetus for exploration of MR directed, cell specific therapies for both women and men in order to improve ischemic heart disease outcomes.

## Introduction

Ischemic heart disease is a leading cause of mortality and morbidity for both men and women (Murray et al., 2012; Wong, 2014). Although the burden of ischemic heart disease has steadily decreased in higher-income countries over the past 25 years, the combination of population growth and aging has led to a 35% increase in deaths from ischemic heart disease globally since 1990 (Moran et al., 2014). Ischemic heart disease is most commonly associated with major coronary vessel atherosclerotic occlusion eventuating in myocardial infarction (MI), though impaired microvascular function is increasingly implicated as an underlying mechanism, especially in women (Maric-Bilkan et al., 2016; Mehta et al., 2016). While survival following a sudden cardiac event has improved, the impact of chronic cardiovascular disease post-MI is increasing. Despite new therapeutic interventions significantly improving acute MI survival, the probability of mortality within 5 years after a first MI remains at approximately 50% (Mozaffarian et al., 2015). Women are especially vulnerable to premature death within 1 and 5 years post-MI regardless of age (Mehta et al., 2016), and are more likely to develop heart failure (Leening et al., 2014). A new generation of novel molecular candidate targets for optimal therapeutic interventions in men and women with ischemic heart disease is urgently required. Mineralocorticoid receptor (MR) directed treatments have emerged as a valuable therapeutic approach in this setting (Buonafine et al., 2018). This review focuses on the clinical/pre-clinical evidence relating to MR influence in the myocardium, and the therapeutic potential for MR antagonists in treating or preventing cardiac ischemia and reperfusion injury in both men and women.

## Mineralocorticoid Receptor Activation and Cardiac Ischemic Injury

The MR is a steroid hormone receptor present in many cell types within the myocardium (Young and Clyne, 2021). MR activation by endogenous ligands aldosterone and cortisol (corticosterone in rodents) is conveyed by a combination of rapid (within minutes) “non-genomic” MR signaling pathways and “genomic” gene transcription and protein synthesis over hours-days (Alzamora et al., 2000; Hayashi et al., 2008; Nolly et al., 2014; Hermidorff et al., 2017; Ong and Young, 2017). The MR was initially recognized for its role in sodium and water homeostasis which occurs *via* direct regulation of sodium, potassium, and other electrolyte handling proteins in the distal nephron. Extensive experimental and clinical studies have highlighted the direct detrimental impact inappropriate MR activation has on the cardiovascular system (Young and Rickard, 2015).

Increased activation of MR *via* inappropriately elevated mineralocorticoid levels or tissue injury are associated with cardiovascular comorbidity and structural remodeling, including fibrosis and myocardial hypertrophy, thereby predisposing the ischemic heart to poor outcomes (Young and Clyne, 2021). MR antagonists have proven benefit for patients with all cause heart failure, with heart failure post-MI and in patients with moderate heart failure, and show potential for patients with heart failure with preserved ejection fraction (HFpEF) (Pitt et al., 1999; Pitt et al., 2003; Markowitz et al., 2012; Miller and Howlett, 2015). Clinical studies (Table 1) indicate that MR antagonists are underutilized and can provide specific benefit for patients with acute MI and high aldosterone levels (Beygui et al., 2009; Rao et al., 2013; Wong et al., 2021). Serum biomarkers for collagen turnover within the RALES and EPHESUS trials indicated MR suppression limits structural remodeling of the extracellular matrix in all cause heart failure and post-MI. However, clinical trials of MR antagonists also suggest that MI classification, heart failure status and timing of MR antagonist administration remain important factors in determining post-MI cardiovascular outcomes (Bulluck et al., 2019; Chen et al., 2021; Mares et al., 2022). The ALBATROSS trial concluded that early MR antagonist administration initiated within 72 h post-MI and prior to the onset of heart failure did not improve patient outcomes and survival six months post-MI (Beygui et al., 2016). In contrast, data from larger cohort populations in combination with results of the REMINDER trial indicated that MR antagonist use reduces the rate of cardiovascular related death irrespective of heart failure status (Beygui et al., 2018). Similarly, the MINIMISE trial reported mixed outcomes in acute ST-segment elevation myocardial infarction (STEMI) patients when MR antagonist was administered immediately prior to reperfusion (Bulluck et al., 2019). In this study infarct size at 3 months was unaffected, while left ventricular remodeling was reduced by MR antagonist therapy. This relative protection from structural remodeling prior to the onset of heart failure post-MI may be key to the benefits observed with MR antagonist administration. With the ongoing development of non-steroidal MR antagonists, additional trials will be required to determine their efficacy in different patient cohorts over an extended period post-MI to improve outcomes.

**TABLE 1:** Summary of clinical trials assessing MR antagonist intervention outcomes.

Disease	Intervention	Outcomes	References
Heart failure	Spironolactone	↓ deaths, ↓ heart failure hospitalization, improved heart failure symptoms	Pitt et al. (1999)
Heart failure post-MI	Eplerenone	↓ deaths/cardiovascular deaths, ↓ cardiovascular deaths and hospitalization	Pitt et al. (2003)
Post-MI	Spironolactone (at reperfusion)	no benefits (vs. standard therapy)	Beygui et al. (2016)
Post-STEMI	MR antagonist (meta-analysis)	↓ all-cause deaths	Beygui et al. (2018)
Post-STEMI	Spironolactone (at reperfusion)	no effect on MI size, improved LV EDV and ESV	Bullock et al. (2019)
Post-MI	MR antagonist (meta-analysis)	↓ all-cause deaths, ↓ cardiovascular event incidence	Chen et al. (2021)
Post-STEMI	MR antagonist (meta-analysis)	↓ all-cause deaths, ↑ LV ejection fraction	
MI, myocardial infarction; STEMI, ST-elevation myocardial infarction; LV, left ventricle; EDV, end-diastolic volume; ESV, end-systolic volume; ↑, increase; ↓, decrease			

## Ischemia, Reperfusion, and the Mineralocorticoid Receptor

The mechanisms that underlie the tissue response to injury during ischemia/reperfusion and the progression to cardiac dysfunction have been studied extensively (Davidson et al., 2019). Interruption of coronary flow to the myocardium impairs cardiomyocyte steady-state metabolism, ultimately leading to dysfunction, arrhythmias, and cell death. The cellular response involves a complex series of events during ischemia can lead to cross-sarcolemmal ion imbalance, activation of stress-responsive intracellular signaling pathways and disruption of critical metabolic processes that contribute to the cardiac pathology. Reperfusion of the myocardium is hence essential to salvage viable myocardium, though this in itself can exacerbate the demise of “at risk” cardiomyocytes (Heusch, 2020). The loss of myocardium culminates in fibrosis and scarring, disrupting normal conduction pathways, which can promote vulnerability to arrhythmia and increase myocardial stiffness. Compensatory hypertrophic growth of the surviving myocardium post-MI maintains functional capacity in the short term, but ultimately the myocardium is unable to compensate for increased wall pressures leading to failure of myocardial pump function and death.

Preclinical and clinical studies further indicate aldosterone excess is a damage provocateur in the ischemic context (Tables 1 and 2). Stimulation of MR by cortisol or aldosterone increase infarct size in *ex vivo* rat hearts even at low doses (Mihailidou et al., 2009), *via* mechanisms that are at least partly attributable to greater cardiomyocyte apoptotic vulnerability. Cardiomyocytes demonstrate aldosterone-induced apoptosis *via* rapid activation of calcineurin and NADPH oxidase/apoptosis signal-regulating kinase one signaling complexes (ASK1) (Mano et al., 2004; Hayashi et al., 2008). The activated MR also mediates upregulation of Ca^2+^ influx that augments cardiomyocyte apoptosis (Ferron et al., 2011). These detrimental actions are exacerbated by MR potentiation of reactive fibrotic remodeling (Brilla and Weber, 1992; Brilla, 2000; Rickard et al., 2009). Exposure of rodents to exogenous mineralocorticoids upregulates transcription of genes underlying extracellular matrix turnover and cardiac remodeling signaling cascades (Rude et al., 2005; Tsai et al., 2013). Together, these findings highlight the potential for MR inhibition to minimize myocardial remodeling following ischemia/reperfusion injury *via* both genomic and potentially non-genomic pathways.

**TABLE 2:** Summary of MR and sex-specific modulation of cardiac structure and function.

Model	Treatment	Intervention	Animal	Sex	Major findings			References
					Fibrosis	Inflammation	Function	
ER modulation								
WT	Aldo/salt	+ERα +ERβ	Rat	F only	↓ perivascular	↓ OPN		Vasan et al. (2004)
Cardiomyocyte-specific ERα overexpression		MI	Mouse	M vs. F	↓ LV (F only)	↑ p-JNK (F only)		Westerman et al. (2016)
Cardiomyocyte-specific ERβ overexpression		Coronary artery ligation	Mouse	M vs. F	↓remote LV (M only)		↑ ejection fraction ↑ diastolic function (F = M)	Kanashiro-Takeuchi et al. (2009)
ERβ deficient	DOC/salt		Mouse	M vs. F	↑ LV (F vs. M)			Gurgen et al. (2011)
MR modulation								
WT	Eplerenone	MI	Rat	M vs. F	↓ LV (F only)		↑ ejection fraction (F only)	Usher et al. (2010)
ERβ knockout	DOC/salt		Mouse	M vs. F	↑ LV (F only)			Lombès et al. (1995)
WT	DOC/salt +/- mTOR-I		Mouse	M vs. F	↑ LV (F only)	↓ (F only)	↑ ejection fraction (M only)	Grohe et al. (1997)
WT	Chronic NO deficiency (*in vivo*)	Acute I/R (*ex vivo*)	Mouse	M vs. F	↑ LV (M = F)	↑ (M = F)	↓ systolic function (F only)	Usher et al. (2010)
Cardiomyocyte-specific MR knockout	Chronic NO deficiency (*in vivo*)	Acute I/R (*ex vivo*)	Mouse	WT vs. KO	↓ LV (KO only)	↓ (KO only)	↑ systolic function (KO only)	Usher et al. (2010)
WT, wild type; Aldo/salt, aldosterone/salt treatment; ERα, estrogen receptor alpha; ERβ, estrogen receptor beta; F, female; M, male; OPN, osteopontin; MI, myocardial infarction; LV, left ventricle; p-JKN, phosphorylated c-Jun N-terminal kinase; DOC/salt, deoxycorticosterone/salt; mTOR-I, mammalian target of rapamycin inhibition; MR, mineralocorticoid receptor; NO, nitric oxide; I/R, ischemia/reperfusion; KO, knockout; ↑, increase; ↓, decrease; = , equal; +, activation; -, inhibition								

### Pre-Ischemic Regulation of Mineralocorticoid Receptor Activity

Many studies have assessed the conditioning capacity of pharmacological agents to minimize injury when administered prior to the ischemic insult. Both long-term and acute inhibition of MR prior to an ischemic event has been shown to be beneficial. Spironolactone administered to rats for 1 month minimized ischemic contracture in isolated hearts subjected to 25 min low-flow ischemia (Rochetaing et al., 2003), indicating a Ca^2+^-dependent mechanism underlying greater myocardial tolerance to the ischemic challenge. During reperfusion, hearts from rats receiving spironolactone exhibited greater functional recovery and less ventricular arrhythmias. Similarly, hearts perfused with 1 μM eplerenone immediately prior ischemia exhibited improved functional recovery in reperfusion and reduced infarct size (Chai et al., 2005). Paradoxically, aldosterone administered prior to ischemia has also been shown to significantly improve contractile function in reperfusion (Yoshino et al., 2014). This observation appears to be mediated by an MR-independent mechanism, for example *via* rapid activation of p38-MAPK which is an important mediator of ischemic preconditioning (Bassi et al., 2008; Bell et al., 2008).

### Targeting Reperfusion Injury—A Role for Pharmacological Regulation of the Mineralocorticoid Receptor

Studies administering pharmacological agents prior to an ischemic insult have both provided considerable insight into the mechanisms of ischemia/reperfusion and identified numerous conditioning agents that could benefit patients undergoing cardiac surgery (Venugopal et al., 2009). However, the practical application of such agents in the clinical setting of a sudden, major ischemic event is very limited and the development of cardioprotective agents that can be administered at the time of reperfusion is a top priority.

Early studies showed that treatment with MR antagonists post-MI had little or no effect on the progression of infarct-healing but can prevent development of reactive fibrosis in the viable rodent myocardium (Delyani et al., 2001; Mill et al., 2003). However, in subsequent studies MR antagonists were found to be functionally beneficial following *in vivo*-MI in rodents for reducing fibrosis of viable myocardium, abrogating increases in left ventricular end diastolic pressure and left ventricular end diastolic volume, and maintaining left ventricular function (Cittadini et al., 2003; Fraccarollo et al., 2003; Fraccarollo et al., 2005). Eplerenone administered post-MI also reduces the onset and progress of cardiac tissue fibrosis (myocardial and aortic), enhances left ventricle ejection fraction and cardiac output, and limits left ventricle systolic area and weight independently of blood pressure (Masson et al., 2004; Wang et al., 2004). Thus, the timing of MR antagonist therapy is key to the functional and gene expression outcome *in vivo*. In addition to fibrotic and functional outcomes, MR blockers improve neovascular formation and reduce thinning and dilation of infarcted myocardial walls at early (3 days) and late (7 weeks) time points in experimental rodents. This action underpins the improvement in ventricular wall function and is associated with transient up-regulation of monocyte chemoattractant protein 1 (MCP-1), early monocyte and macrophage infiltration and expression of tumour necrosis factor alpha (TNFα) (Fraccarollo et al., 2008). These beneficial outcomes are mirrored in transgenic mice lacking the MR in cardiomyocytes, underscoring the central role of the receptor for the cardiomyocyte response to ischemia/reperfusion and are discussed further below (Fraccarollo et al., 2011).

Investigation of the effects of MR suppression on global remodeling of the left ventricular chamber have reported variable findings. In a rat model of MI, spironolactone showed no benefit for reducing left ventricle cardiac chamber mass index and wall thickness despite reduced cardiomyocyte cross sectional area and less fibrosis (Enomoto et al., 2005). Whereas other studies show reversal of left ventricle dilation and dysfunction with MR blockade post-MI *via* mechanisms that include suppression of NADPH oxidase and mitochondrial superoxide production (Matsumoto et al., 2004).

### Genetic Manipulation of Mineralocorticoid Receptor Signaling Reveals Ischemic Vulnerability

To more precisely understand the specific myocardial cell types involved in mediating MR-dependent adverse and beneficial outcomes, targeted genetic manipulation studies have proved to be particularly informative (Table 2). Initial MR deletion mouse models were homologous MR knockout, which displayed neonatal lethality due to sodium wasting (Berger et al., 1998). Subsequent studies focused on genetic manipulation of MR in specific cell types, including macrophages, vascular smooth muscle, endothelial and cardiomyocytes (Young and Rickard, 2015). The cardiomyocyte MR knockout mouse (myo-MRKO) has demonstrated a range of novel and important actions of the MR in the regulation of the tissue response to cardiac ischemia including promoting an appropriate wound healing response in the infarct zone, enhanced neovascularization of the scar and maintenance of the microvascular capillary network, which together support cardiac functional recovery and long term viability (Fraccarollo et al., 2011).

Our studies subjecting *ex vivo* myo-MRKO hearts to an acute IR challenge demonstrated improved contractile functional recovery and lower vulnerability to arrhythmias compared with wild-type controls (Bienvenu et al., 2015). This was associated with reduced expression of the sodium-hydrogen exchanger (NHE-1) and reduced CaMKII autophosphorylation, both of which are predicted to minimize cardiomyocyte Na^+^ and Ca^2+^ loading and suppress cardiac dysfunction in IR (Bell et al., 2014; Mattiazzi et al., 2015; Shattock et al., 2015). Genetic inhibition of CaMKII combined with MR antagonist treatment improved functional recovery and reduced diffuse fibrosis, suggesting that targeting both pathways can potentially improve contractile performance and reduce arrhythmic activity (Driessen et al., 2020). In a chronic model of MI, suppressing cardiomyocyte MR signaling was also beneficial. Targeted ablation of cardiomyocyte MR did not affect infarct size *in vivo*, yet morphological changes were minimized, and ventricular function better maintained in the subsequent 8 weeks post-MI (Fraccarollo et al., 2011). Expression of genes associated with hypertrophy, stiffness and fibrosis was lower in surviving myocardium from myo-MRKO mice and myocardial/mitochondrial superoxide production was diminished (Fraccarollo et al., 2011). The authors concluded that a suppressed NFκB-mediated inflammatory response was key to minimizing apoptosis and enhancing healing in these mice.

**FIGURE 1:**
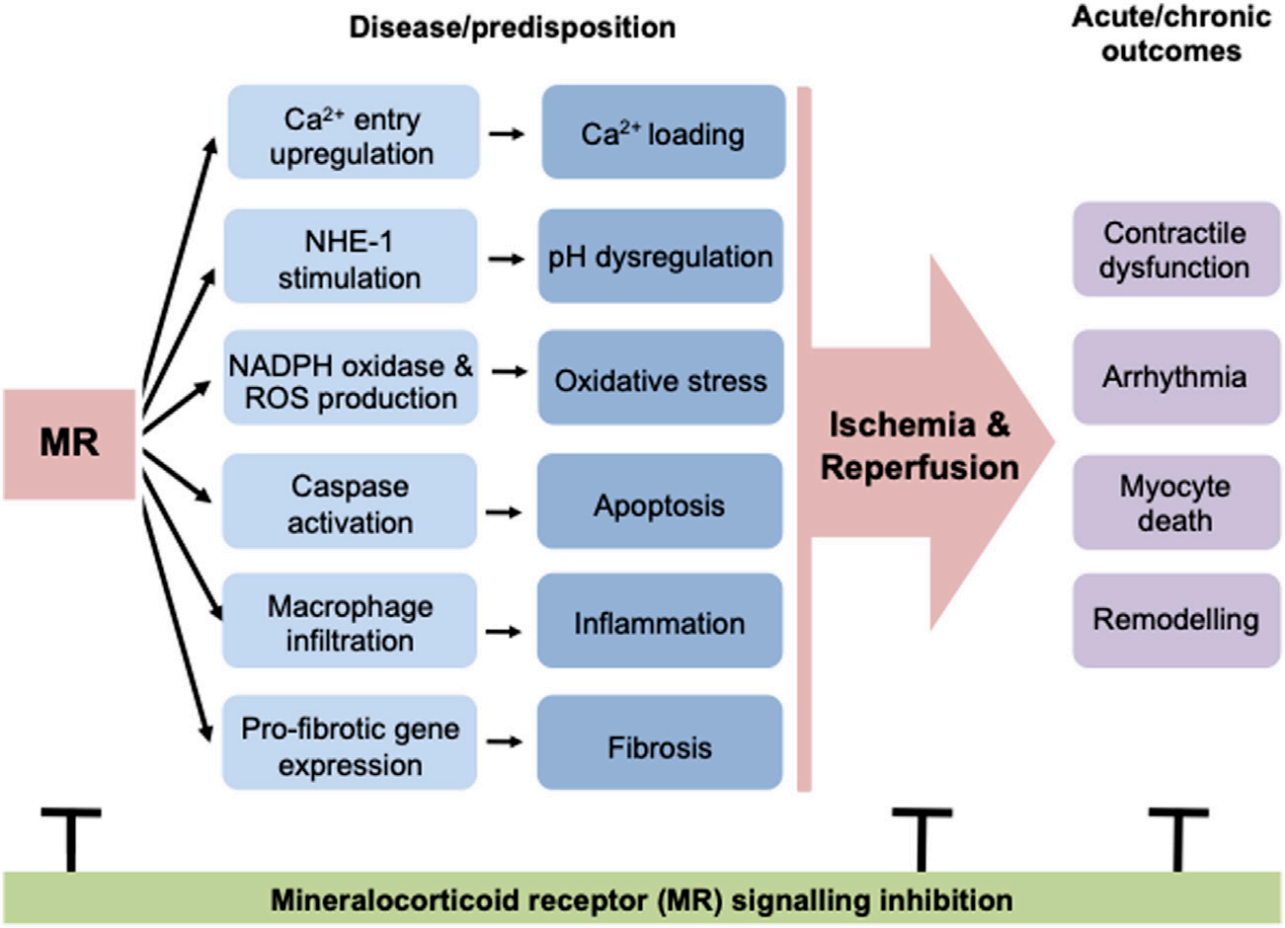
MR activation leads to many detrimental changes in the heart, including cardiomyocyte cell death, inflammation and fibrosis. In addition to these structural changes, MR signaling leads to stimulation of calcium and sodium flux in cardiomyocytes, predisposing these cells to calcium loading and pH dysregulation. Overall these modulations of basal cardiac structure and function predispose the heart to ischemic events and result in worse outcomes after ischemia/reperfusion, increasing the incidence of contractile dysfunction and arrhythmia and also increasing cell death and consequently reactive fibrosis. The negative impacts of MR activation at a structural and functional level can be abrogated at early and late timepoints, both before and after an ischemic event. MR, mineralocorticoid receptor; NHE-1, sodium hydrogen exchanger-1; ROS, reactive oxygen species.

Deletion of MR in other specific cell types of the myocardium (vascular smooth muscle, endothelial, macrophage cells) demonstrate cell-specific regulation of myocardial injury and repair pathways in a manner that would be predicted to confer protection in the ischemic setting (Bienvenu et al., 2012; Gueret et al., 2016; Fraccarollo et al., 2019). This demonstrates the importance of MR signaling across numerous cardiac cell types in the ischemic context. This may be critical to determining how targeting MR signaling may be optimized post-MI. Further studies are hence required to explore in greater detail how MR-mediated regulation of different cell populations within the heart interact to determine the functional and morphological responses to IR (Figure 1).

## Mineralocorticoid Receptor Signaling, Sex Specificity and Cardioprotection

The incidence of cardiovascular disease (and more specifically ischemic heart disease) differs between the sexes, with earlier onset in men and increasing incidence in women post-menopause (Leening et al., 2014). Men and women exhibit differences in symptom presentation, efficacy of diagnosis and response to interventions (McSweeney et al., 2016). More specifically, in relation to myocardial infarction, sex-specific differences in pathophysiological mechanisms and outcomes is now being emphasized (Mehta et al., 2016). Women are more likely to develop heart failure after an acute MI, underscoring the need for new strategies and therapies to adequately address long term MI recovery in both sexes (Westerman and Wenger, 2016).

Most clinical studies assessing suppression of MR signaling have involved predominantly male cohorts, and even though data are adjusted to consider sex as a covariate, differences in clinical outcomes between men and women may not be observed (Pitt et al., 2003; Kanashiro-Takeuchi et al., 2009; Nicolaou, 2021). Moreover, serum aldosterone levels have been strongly correlated with left ventricular mass in females, but not males even when serum aldosterone levels are not elevated and the MR inhibitory effect of progesterone is taken into consideration (Vasan et al., 2004). Given the importance of cardiac hypertrophy and serum aldosterone levels as independent cardiovascular disease risk factors, this observation indicates that some of the differences observed for males and females in the clinical setting may also have an MR-dependent mechanism. Endothelial MR expression has been shown to be higher in female vessel wall, when compared with males (Faulkner et al., 2019). This observation reflects the fact that females have higher circulating levels of progesterone which is a natural antagonist of the MR and estrogen acting *via* the ERα inhibits MR activation. Higher aldosterone levels and potentially tissue expression of MR in females is likely to be compensatory in premenopausal women.

Steroid hormone ligands for both receptors derive from the same cholesterol-derived progestogen pre-cursors. Relative levels of corticosteroids and sex steroids are hence closely linked. It is well established that estrogen receptors (ER) and MR are co-expressed in cardiomyocytes, fibroblasts and vascular cells (Lombès et al., 1995; Grohe et al., 1997), and that these can modulate cardiac structure and function in settings of IR. Transgenic animal models have shown that female hearts overexpressing ERα receptor subtype are relatively protected from MI-induced fibrosis and exhibit improved neovascularization (Mahmoodzadeh et al., 2014). Overexpression of the ERβ receptor subtype improves survival and cardiac function post-MI in both sexes, due in part to reduced disturbance of cardiomyocyte intracellular Ca^2+^ store management (Bell et al., 2013). In male hearts specifically, functional improvement was related to a lower level of cardiac fibrosis highlighting the role of ERβ in post-infarct remodeling (Schuster et al., 2016). In rodent models activation of both ERα and ERβ protects against the detrimental blood pressure, fibrotic and hypertrophic effects of mineralocorticoid excess (Arias-Loza et al., 2007). For example, deoxycorticosterone/salt-induced cardiovascular damage is reduced in females with intact ER signaling. Whereas genetic ablation of ERβ signaling produces a differential response to mineralocorticoid excess-induced cardiac fibrosis associated with mTOR (mammalian target of rapamycin) activation in both male and female mice (Gurgen et al., 2011; Gurgen et al., 2013). Moreover, the transcriptional activity of vascular MR can be inhibited by ERα, suggesting that MR/ER interactions contribute to the mechanisms of sex differences in cardiac MR activity (Barrett Mueller et al., 2014; Biwer et al., 2021). Rapid aldosterone effects may also be medicated by the G-protein coupled ER, though evidence of direct aldosterone binding remains contentious (Rossol-Haseroth et al., 2004; Michea et al., 2005; Gros et al., 2011; Gros et al., 2013). An important consideration moving forward will be developing a more detailed understanding of the nature of the interaction of MR with all ER subtypes in influencing genomic and rapid activation signaling pathways (Funder, 2011).

Experimental studies that specifically address sex-difference in MR involvement in cardiac ischemia are limited but some important leads have been reported. Suppression of MR signaling in rodents by eplerenone administered after MI has been shown to achieve more effective attenuation of left ventricular end diastolic volume enlargement in female compared to male hearts (Kanashiro-Takeuchi et al., 2009). Additionally, LV ejection fraction was increased in female hearts. Transcriptome analysis revealed that for female hearts, eplerenone reversed transcriptional responses for 19% of down regulated genes and for 44% of up-regulated genes, whereas only 4% of genes up-regulated in male hearts responded to eplerenone. These data indicate that MR blockade may preferentially reduce structural and functional changes in female hearts through initiation of specific transcriptional responses (Kanashiro-Takeuchi et al., 2009).

Nitric oxide (NO) signaling is known to be important in mediating cardioprotection (Griffiths et al., 2021)—and there is clear evidence of MR involvement in NO modulation in endothelial and other cell types. Heart failure is associated with low levels of bioavailable NO, and clinical evidence indicates sex differences in NO mediated responses (Zamani et al., 2015). In addition, an imbalance of NO levels has been shown to be a key component in the development of heart failure with preserved ejection fraction in males, but not necessarily females (Schiattarella et al., 2019; Tong et al., 2019). There is extensive experimental evidence to indicate that in female cardiac disease states, NO production and involvement in ROS modulation is important (Murphy et al., 2011; Casin et al., 2018). Targeting NO bioavailability is therefore considered an attractive target for sex-specific therapies in heart failure, but less is known about possible benefits in ischemic disease states. Given that there is evidence for an MR-NO signaling link in endothelial cells, regulating the MR has the potential to optimize NO conditions and support cardiomyocyte protection during cardiac ischemia/reperfusion (Jia et al., 2016; Victorio et al., 2016). Our experimental findings have shown that after chronic *in vivo* treatment with an NO-synthase inhibitor, male and female cardiomyocyte specific MR-KO mice exhibit different cardiac *ex vivo* responses. While female hearts showed MR-dependent abrogation of NO-deficiency induced ischemic injury (reperfusion arrhythmia, diastolic dysfunction and impaired contractile recovery), male animals did not exhibit a similar MR-NO deficiency response interaction (Bienvenu et al., 2017). More recently, it has been shown that ERα mediated NO production can abrogate the detrimental impact of MR activation in the microvasculature of obese female rodents, highlighting the interplay between MR and ER in the endothelium (Biwer et al., 2021). Further work is required to define the nature of the signaling relationship between MR, ER, and NO in order to effectively exploit this signaling axis for therapeutic outcomes in tissue injury due to ischemia and reperfusion.

## Conclusion

In this review, the accumulating clinical and preclinical data indicating important involvement of MR signaling in mediating both acute and longer term cardiac ischemic damage have been considered. Whilst a range of cardiac cell types are involved (macrophages, endothelial, and vascular smooth muscle), the cardiomyocyte-specific mineralocorticoid signaling pathways appear to be key. Evidence supports a role for increased aldosterone levels and MR activation in mediating sex-specific aspects of ischemic vulnerability through MR-ER receptor interactions providing important insights into ischemic heart disease in women. While early clinical trials of MRA showed equal protection for heart failure in females as well as males, discrepancies remain between translation of experimental outcomes and observed clinical sex-differences in the etiology and diagnosis of heart failure. Thus, there is considerable impetus for exploration of mineralocorticoid-directed, cell-specific therapies for both women and men in order to improve ischemic heart disease outcomes. Specific, ongoing challenges involve dissecting the integrative nature of the MR-ER-NO signaling axis so that sex-specific therapies can be identified to address both acute and chronic phases of ischemic injury. In this setting, preclinical mechanistic investigations of cell specific MR interactions with pathways regulating ischemia injury have considerable capacity to inform ongoing clinical studies.
